# Intrinsic Sinus Node/Atrioventricular Node Dysfunction Requiring Pacemaker Implantation: Role of Former Professional Sport Activity

**DOI:** 10.3390/jcm13010203

**Published:** 2023-12-29

**Authors:** Sergei Bondarev, Evgeny Achkasov, Alessandro Zorzi, Alexandr Safaryan, Francesca Graziano, Alexey Sizov

**Affiliations:** 1 Department of Cardiac, Thoracic and Vascular Sciences and Public Health, University of Padova, 35128 Padova, Italy; sergei.bondarev@unipd.it (S.B.);; 2 Department of Sports Medicine and Medical Rehabilitation, Sechenov First Moscow State Medical University (Sechenov University), 119991 Moscow, Russia; 3 Cardiology Department, St. Alexius Hospital, 119071 Moscow, Russia

**Keywords:** athlete’s heart, atrioventricular block, pacemaker, sports cardiology, sinus bradycardia

## Abstract

Background: Sinus bradycardia and first degree or second degree Mobitz type I atrioventricular (AV) block in an athlete are considered adaptive and reversible phenomena; however, some evidence suggests that they may persist after detraining and become pathological. The aim of the study was to investigate the characteristics of a group of former professional athletes who required pacemaker (PM) implantation for intrinsic (idiopathic) sinus node (SN) dysfunction or AV block in comparison to control groups of sedentary individuals. Methods: We included all patients who underwent PM implantation during 2022. Three groups were compared: group 1 including 18 former professional athletes who received a PM for SN dysfunction/AV block in the absence of heart disease; group 2 including the first 20 sedentary individuals without heart disease who underwent PM implantation; and group 3 including all other 323 patients who received PM, the majority with underlying heart diseases. Results: Compared to the non-athlete control group 2, the mean age at diagnosis and at the time of PM implantation of former professional athletes did not show statistically significant differences. However, subgroup analysis revealed significant differences depending on the type of sports discipline: the age at diagnosis and at PM implantation was significantly lower in former endurance athletes than former strength/mixed athletes, control non-athletes, and all other patients. Moreover, former endurance professional athletes exhibited a higher prevalence of second or third degree AV block (78%) as the reason for PM implantation compared to power/mixed athletes (44%). The other clinical characteristics, including echocardiographic parameters, did not differ between former athletes and non-athletes. Conclusions: Former professional endurance athletes with idiopathic SN dysfunction/AV block manifested the disease earlier in the life course compared to former power/mixed athletes and non-athletes. This suggests that bradycardia/AV block caused by intense and prolonged endurance sports may not always be benign and adaptive phenomena.

## 1. Introduction

Prolonged exercise training is associated with the so-called remodeling of the athlete’s heart. From an electrical point of view, two common features of the athlete’s heart are sinus bradycardia and first-degree (rarely second-degree Mobitz type 1) atrioventricular (AV) block [1,2]. The biological explanation for this phenomenon may lie in the physiological adaptation of the heart to exercise that is characterized by a harmonic enlargement of the four heart cavities. Given the high systolic volume provided by those large ventricles, a slower heart rate would be enough to provide sufficient cardiac output and oxygenation of the body at rest. Moreover, a longer interval between atrial and ventricular contraction may allow more time for the large atria to empty. Complete restoration of the function of the sinus (SN) and atrioventricular (AV) nodes in response to physical activity is observed in normal athletes [3]. The electrical changes in response to exercise are not only found in elite professional athletes but also in amateur sportsmen [4].

Traditionally, sinus bradycardia and AV block in the athlete has been attributed to vagal activity and, as such, considered reversible with detraining [1]. However, recent evidence derived from animal studies suggest an alternative hypothesis: exercise would cause a transcriptional remodeling of ion channels’ proteins that translates into the reduced current density of key ionic currents involved in impulse generation and conduction [5,6,7]. Consistent with this hypothesis, Stein et al. demonstrated that under double-pharmacologic blockade of the autonomic nervous system (with atropine and beta-blocker), SN and AVN properties in endurance athletes continued to show differences compared to controls, suggesting that they are related to intrinsic physiology and not to autonomic influences [8].

At variance with parasympathetic stimulation, this transcriptional remodeling may persist long after cessation of the athletic career, potentially adding to age-related intrinsic SN or AV node dysfunction and resulting in a higher incidence of clinically overt sinus bradycardia or AV block requiring pacemaker (PM) implantation in athletes compared to the general population. In a study that compared former professional cyclists with a control group of golfers, the former cyclists showed a lower heart rate at rest, although the two groups did not differ in terms of the current hours of physical exercise per week. On 24 h ambulatory ECG, former cyclists also showed a higher prevalence of profound sinus bradycardia < 40 b.p.m., sinus pauses, and PM implantation (3% versus 0%) [9]. However, the association between sports activity and persistent SN or AV node dysfunction after cessation of the athletic career is still debated.

Another open question is whether the type of sports activity that was practiced would affect the chance of developing pathological SN or AV dysfunction later in the life course. It is known that endurance athletes show more pronounced heart remodeling (both structural and electrical) [3]. However, to date there are no clear data suggesting a higher chance of SA and AV disturbances persisting after detraining in former endurance athletes compared to former athletes practicing strength or mixed disciplines.

The aim of the study was to investigate the characteristics of a group of former professional athletes who required PM implantation for intrinsic (idiopathic) SN dysfunction or AV block in comparison to control groups of sedentary individuals.

## 2. Materials and Methods

This prospective observational study was conducted at the cardiology clinic of the Sechenov University, Moscow (Russia) during 2022. The study included 381 people who received a PM for clinically significant SN disease or AV block according to current European Society of Cardiology recommendations [10]. SN or AVN dysfunction was defined as “idiopathic” in the absence of heart failure, ischemic heart disease, previous or acute myocarditis, systemic connective tissue disease, congenital defect, or cardiomyopathy.

Study group 1 consisted of 18 former elite professional athletes (9 men, 9 women, mean age 69 ± 13 years) who participated in international competitions such as world championship or Olympic Games and received a PM for idiopathic SN disease or AVN block. Of these, 9 (6 men, 3 women, average age 65.0 ± 11.7 years) used to practice endurance sports (cycling N = 4, cross-country skiing N = 2, long-distance running N = 2, rowing N = 1), while the remaining 9 former athletes (4 men and 5 women, average age 73.0 ± 14.3 years) used to practice power or mixed sports (martial arts N = 4, discus thrower N = 1, weightlifting = 2, soccer N = 1, basketball N = 1). Classification of disciplines followed the European Society of Cardiology guidelines on sports cardiology [3]. Former athletes who practiced skill disciplines at low cardiovascular demand were not included in group 1 because of the limited cardiovascular remodeling associated with these kinds of sports.

All 343 patients who received PM implantation in the same year and were not former professional athletes were also included. From these patients, a control group consisting of the first 10 men and 10 women with idiopathic SN dysfunction or AVN block who underwent PM implantation was selected. The remaining 323 patients who received PM in 2022 formed group 3.

Clinical data (history, blood tests, ECG, Holter ECG monitoring, echocardiography) were obtained at the time of device implantation. In particular, echocardiographic measures were performed according to current guidelines [11].

Categorical variables were compared using the chi-square test or Fisher’s exact test, as appropriate, while continuous variables were compared using the rank sum test given the small sample size. The Bonferroni correction was used when performing subgroup analysis. Data were analyzed using Statsoft ver. 13 (Tulsa, OK, USA), and a *p* value < 0.05 was considered significant.

## 3. Results

The main characteristics of the three groups of patients are reported in Table 1.

### 3.1. Former Athletes Group

The mean age at the beginning of sport activity of former professional athletes was 12.8 ± 3.8 years, but former endurance athletes started significantly earlier (11.4 ± 3.8 years) compared to power/mixed disciplines athletes (14.2 ± 3.4 years, *p* < 0.05). Likewise, the mean age at the cessation of the professional career was significantly higher in endurance athletes compared to power/mixed athletes (27.0 ± 6.6 versus 23.0 ± 2.3 years, *p* < 0.05). The hours of exercise/week during the peak of the athletic career were similar between the two subgroups (on average 13 h/week).

The first symptoms possibly attributed to bradycardia (weakness, dizziness, and irregular heartbeat) occurred in athletes at a mean age of 46.7 ± 16.3 years, with no significant differences between endurance and strength athletes. The age of clinical diagnosis of SN dysfunction or AV block was on average 65.0 ± 9.3 years, significantly higher in the power/mixed group (72.4 ± 9.5 years) compared to the endurance group (64.0 ± 7.5 years, *p* < 0.01). Similarly, the age of PM implantation was 68.5 ± 13.0 years, significantly higher in the power/mixed group (73.0 ± 10.3 years) compared to the endurance group (65.0 ± 11.7 years *p* < 0.05). There was also a significant difference in the reason for PM implantation between the two subgroups with a higher prevalence of AV block in endurance athletes (78%) compared to power/mixed athletes (44%). All patients received dual-chamber frequency-adaptive pacemakers (Enitra 6 DR, Effecta DR or Enitra SR).

In all cases, SN or AVN dysfunction were intermittent, resulting in a percentage of stimulation of 5.0 ± 2.3% three months after implantation. Interestingly, the absence of bundle branch block on the ECG in patients with AV block suggests the absence of infra-hissian conduction defects.

### 3.2. Comparison between Athletes and Non-Athletes

The mean age at diagnosis and at the time of PM implantation (Group 1) in former athletes was non-significantly lower than in the control non-athletes (Group 2). However, subgroup analysis showed that the difference between former endurance athletes and control non-athletes was significant (*p* < 0.01) for both age at diagnosis and age at PM implantation, while the differences between the former mixed/power athletes and the controls were not significant. Other clinical characteristics, including ECG and echocardiographic parameters, did not differ between athletes and non-athlete controls.

Group 3 included the remaining 323 patients (182 men, 141 women, mean age 75 years, 0 ± 11.4) who received PM implantation: the majority had various diseases such as a history of myocardial infarction (72%) or stage 2 and 3 arterial hypertension (92%). When comparing athletes with all other patients who underwent PM implantation in 2022 (group 3), similar trends towards a lower age at the time of clinical manifestation of SN/AVN dysfunction and at the time of PM implantation were noted.

## 4. Discussion

**Figure 1:**
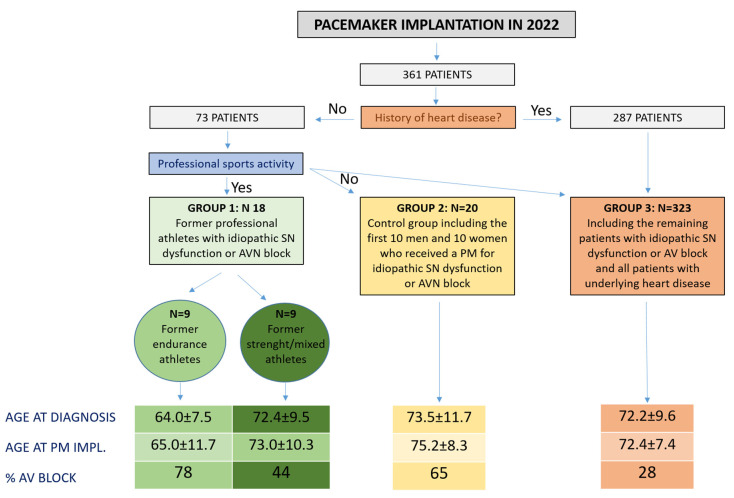
Summary of main study methods and findings.

The study enrolled a cohort of former professional athletes who developed symptomatic SN or AVN dysfunction requiring PM implantation, in the absence of underlying heart disease. The sample was divided into two subgroups: former endurance athletes (cyclists, runners…) and former mixed (team sports) or power (wrestlers, weightlifters…) athletes. The characteristics of this group were compared to that of a control group consisting of nonathletes without heart disease and to a third group including all other patients who received a PM during the study period, the majority with an underlying heart disease. The study methods and main results are summarized in Figure 1.

Although athletes showed an earlier development of symptoms and need for PM implantation compared to control non-athletes, the difference did not reach statistical significance. However, when comparing the two subgroups of former athletes according to the sport discipline, we noted no differences between former power/mixed athletes and the controls, while former endurance athletes developed symptoms and received a PM at a significantly lower age than both power/mixed athletes and controls. Moreover, endurance athletes showed a higher prevalence of second or third degree AVN block as the reason for PM implantation than power/mixed athletes and the controls.

According to current recommendations for interpretation of the athlete’s ECG, sinus bradycardia and first/second-degree Mobitz type 1 AV block are considered common findings, and if they are asymptomatic and normalize during exercise, they do not require further investigations [1,12,13]. Profound sinus bradycardia with a heart rate of 30 or less and second-degree atrioventricular block Mobitz type 2 can also be caused by extremely strenuous exercise [3]. Although such changes are considered benign and reversible, there is limited evidence that former endurance athletes may be more prone to persistent SN/AVN dysfunction later after the cessation of their athletic career [9,14,15,16,17]. According to our study, we also found that a cohort of former professional endurance athletes with intrinsic SN/AVN dysfunction developed symptoms and required PM implantation earlier than former power/mixed athletes and control non-athletes. This suggests that the long-term effect of prolonged sports activity may have caused or anticipated the onset of overt sinus bradycardia/AV block.

It is known that training results in an increased parasympathetic stimulation in athletes and that athletes, particularly those engaged in endurance disciplines, often show sinus bradycardia and first-degree AV block (rarely second-degree type 1 AV block) [1,12,13]. For this reason, sinus bradycardia and AV block have been traditionally attributed to the (reversible) effect of parasympathetic stimulation [15,18]. However, previous small studies showed that dual block of the autonomic nervous system through simultaneous administration of atropine and propranolol do not completely restore SN and AVN function in athletes [7,8]. More recently, animal studies confirmed that the activity of the sinus and AV nodes in athletes are not simply influenced by the autonomic nervous system activity, but that prolonged exercise downregulates the transcription of ion channel proteins [5,6,7]. It may be hypothesized that detraining may not completely reverse the exercise-induced modifications in SN and AVN function favoring the development of symptomatic sinus bradycardia or AV block more often and earlier during the life course than sedentary individuals [19].

Another possible theory linking sports activity to SN and AVN damage involves the direct toxic effects of catecholamines, peroxides, and severe metabolic acidosis [20,21,22,23,24,25]. Stimulation of β-adrenergic receptors of cardiomyocytes with catecholamines in concentrations above physiological levels leads to an increase in adenylate cyclase activity through stimulation of Gs protein synthesis. ATP is subsequently converted to cAMP, which activates a protein kinase that phosphorylates and open Ca^2+^ channels. As a result, the concentration of Ca^2+^ in cardiomyocytes increases. Activation of Ca^2+^-dependent proteases and Na+/Ca^2+^ exchange channels leads to the acceleration of oxidative processes and accumulation of reactive oxygen species and ultimately can lead to necrosis of cardiomyocytes and their apoptosis. This theory may explain why SN/AVN dysfunction occurred earlier in former endurance athletes, who began practicing sports earlier and were exposed to more prolonged and more intense training.

## 5. Study Limitations

The main limitation of the study is the small sample size because we included only elite athletes who used to compete at international level. In particular, comparison between the subgroups of athletes was based on very small numbers, although the differences between them were statistically significant. On the other hand, the lack of statistically significant differences between the entire sample of athletes and controls should be interpreted with caution considering the low statistical power. For these reasons, our preliminary data need to be confirmed by further studies on larger populations.

## 6. Conclusions

In conclusion, the study suggests a possible contribution of former professional endurance sports activity to the earlier development of intrinsic SN disease or AV block requiring PM implantation. According to this finding, we can speculate that sinus bradycardia and AV block in highly trained athletes may not always be an adaptive (benign) and reversible phenomenon but may accelerate the onset of clinically significant SN/AVN dysfunction during the life course. However, we must recognize that these data were obtained in a small sample of patients and that further studies on a larger population are needed to confirm this hypothesis.

## Figures and Tables

**Table 1:** Characteristics of patients who underwent PM implantation in 2022. Group 1: former professional athletes with idiopathic SN disease or AV block. Group 2: non-athlete controls with idiopathic SN disease or AV block. Group 3: all other patients.

Options	Group 1			Group 2 N = 20	Group 3 N = 323	*p* *
	Former Power/Mixed Athletes N = 9	Former Endurance Athletes N = 9	All Former Athletes N = 18			
Age, years	73.0 ± 10.3	65.0 ± 9.3	69.2 ± 11.4	75.2 ± 8.3	75.0 ± 11.4	0.07
Age at the beginning of sport activity, years	14.2 ± 3.4	11.4 ± 3.8	12.8 ± 3.8	-	-	-
Professional sport practice, years	11.5 ± 4.5	13 ± 7.5	12.3 ± 6.2	-	-	-
Training hours per week at peak of career	13.0 ± 3.4	12.6 ± 3.0	12.8 ± 3.0	-	-	-
**Clinical history**						
Atrial fibrillation, %	4 (44)	5 (56)	9 (50)	8 (40)	209 (65)	0.02
Age at onset of symptoms, years	45.8 ± 14.7	48.0 ± 17.0	46.7 ± 16.3	53.4 ± 6.1	65.3 ± 8.1	<0.01
Age at diagnosis, years, years	72.4 ± 9.5	64.0 ± 7.5	67.0 ± 9.3	73.5 ± 11.7	72.2 ± 9.6	0.11
Age at PM implantation, years	73.0 ± 10.3	65.0 ± 11.7	68.5 ± 13.0	75.2 ± 8.3	72.4 ± 7.4	0.08
Complaints of weakness, %	5 (56)	6 (66)	11 (61)	20 (100)	321 (99)	<0.001
Complaints of shortness of breath, %	5 (56)	7 (78)	12 (67)	16 (80)	264 (82)	0.06
Sinus node dysfunction, %	5 (56)	2 (22)	7 (39)	7 (35)	232 (72)	<0.001
AVB of second or third degree, %	4 (44)	7 (78)	11 (61)	13 (65)	90 (28)	<0.001
**ECG**						
PR interval ms	231.4 ± 5.0	230.5 ± 4.5	230.0 ± 6.0	190 ± 19	220 ± 18	0.25
QTc interval, ms	437.5 ± 29.0	441.3 ± 22.0	438.7 ± 26.0	433 ± 18	440 ± 25	0.72
Left His bundle branch block, %	0	0	1 (5.5)	0	113 (35)	#
Right His bundle branch block, %	0	0	0	2 (10)	145 (45)	#
**Echocardiography**						
Right atrium minor axis, cm	3.9 ± 0.9	3.8 ± 0.7	3.8 ± 0.8	3.8 ± 0.4	4.5 ± 07	0.02
Right atrium major axis, cm	5.4 ± 1.9	5.2 ± 1.2	5.3 ± 1.7	5.2 ± 1.2	5.5 ± 1.2	0.23
Left atrium antero-posterior dimension, cm	4.1 ± 0.6	4.7 ± 1.0	4.3 ± 0.8	4.2 ± 0.4	5.0 ± 0.5	0.02
Left ventricular end-diastolic dimension, cm	4.5 ± 0.9	5.1 ± 1.5	4.7 ± 1.1	4.8 ± 0.4	5.6 ± 1.3	0.01
Left ventricular end-systolic dimension, cm	2.9 ± 0.5	3.3 ± 1.2	3.1 ± 0.8	3.0 ± 0.3	4.5 ± 0.9	0.01
Interventricular septal thickness	1.2 ± 0.1	1.2 ± 0.1	1.2 ± 0.1	1.2 ± 0.08	1.3 ± 0.07	0.82
Proximal right ventricle outflow tract, cm	3.8 ± 1.1	3.4 ± 1.0	3.7 ± 1.0	3.9 ± 0.11	3.7 ± 0.21	0.53
Distal right ventricle outflow tract, cm	2.6 ± 0.3	2.9 ± 0.4	2.7 ± 0.4	2.9 ± 0.3	3.3 ± 0.5	0.04
Aortic root diameter, cm	3.1 ± 0.5	3.6 ± 0.4	3.4 ± 0.5	3.3 ± 0.4	3.8 ± 0.6	0.26
E/A	0.8 ± 0.15	0.7 ± 0.16	0.7 ± 0.15	0.7 ± 0.03	1.2 ± 0.06	<0.01
T dec, ms	298.0 ± 41.2	291.0 ± 41.0	296.0 ± 40.4	306 ± 24	286 ± 31	0.42

AVB = atrioventricular block, PM = pacemaker; PVBs = premature ventricular beats. * *p* values for comparison among the entire group 1, group 2, and group 3; # chi-square test could not be calculated because of cells with 0 value.

## Data Availability

The data presented in this study are available upon request from the author Bondarev Sergey. The data are not publicly available because it is collected and stored in a local archive without access to a single network.

## References

[1] Sharma S., Drezner J.A., Baggish A., Papadakis M., Wilson M.G., Prutkin J.M., La Gerche A., Ackerman M.J., Borjesson M., Salerno J.C. (2018). International recommendations for electrocardiographic interpretation in athletes. Eur. Heart J..

[2] Huttin O., Selton-Suty C., Venner C., Vilain J.-B., Rochecongar P., Aliot E. (2018). Electrocardiographic patterns and long-term training-induced time changes in 2484 elite football players. Arch. Cardiovasc. Dis..

[3] Pelliccia A., Sharma S., Gati S., Bäck M., Börjesson M., Caselli S., Collet J.P., Corrado D., Drezner J.A., Halle M. (2021). 2020 ESC Guidelines on sports cardiology and exercise in patients with cardiovascular disease. Eur. Heart J..

[4] Bjørnstad H., Storstein L., Meen H.D., Hals O. (1994). Ambulatory electrocardiographic findings in top athletes, athletic students and control subjects. Cardiology.

[5] Mesirca P., Nakao S., Nissen S.D., Forte G., Anderson C., Trussell T., Li J., Cox C., Zi M., Logantha S. (2021). Intrinsic Electrical Remodeling Underlies Atrioventricular Block in Athletes. Circ. Res..

[6] Bidaud I., D’Souza A., Forte G., Torre E., Greuet D., Thirard S., Anderson C., Chung You Chong A., Torrente A.G., Roussel J. (2021). Genetic Ablation of G Protein-Gated Inwardly Rectifying K^+^ Channels Prevents Training-Induced Sinus Bradycardia. Front. Physiol..

[7] D’Souza A., Sharma S., Boyett M.R. (2015). CrossTalk opposing view: Bradycardia in the trained athlete is attributable to a downregulation of a pacemaker channel in the sinus node. J. Physiol..

[8] Stein R., Medeiros C.M., Rosito G.A., Zimerman L.I., Ribeiro J.P. (2002). Intrinsic Sinus and Atrioventricular Node Electrophysiologic Adaptations in Endurance Athletes. JACC.

[9] Baldesberger S., Bauersfeld U., Candinas R., Seifert B., Zuber M., Ritter M., Jenni R., Oechslin E., Luthi P., Scharf C. (2008). Sinus node disease and arrhythmias in the long-term follow-up of former professional cyclists. Eur. Heart J..

[10] Glikson M., Nielsen J.C., Kronborg M.B., Michowitz Y., Auricchio A., Barbash I.M., Barrabés J.A., Boriani G., Braunschweig F., Brignole M. (2021). 2021 ESC Guidelines on cardiac pacing and cardiac resynchronization therapy. Eur. Heart J..

[11] Lang R.M., Badano L.P., Mor-Avi V., Afilalo J., Armstrong A., Ernande L., Flachskampf F.A., Foster E., Goldstein S.A., Kuznetsova T. (2015). Recommendations for cardiac chamber quantification by echocardiography in adults: An update from the American Society of Echocardiography and the European Association of Cardiovascular Imaging. Eur. Heart J. Cardiovasc. Imaging.

[12] Corrado D., Pelliccia A., Heidbuchel H., Sharma S., Link M., Basso C., Biffi A., Buja G., Delise P., Gussac I. (2010). Recommendations for interpretation of 12-lead electrocardiogram in the athlete. Eur. Heart J..

[13] D’Ascenzi F., Zorzi A., Corrado D., Thompson P., Fernandez A. (2018). Chapter 5: Electrophysiologic Adaptation to Exercise and Management of Arrhythmias in the Athlete. Exercise and Sports Cardiology.

[14] Graziano F., Juhasz V., Brunetti G., Cipriani A., Szabo L., Merkely B., Corrado D., D’Ascenzi F., Vago H., Zorzi A. (2022). May Strenuous Endurance Sports Activity Damage the Cardiovascular System of Healthy Athletes? A Narrative Review. J. Cardiovasc. Dev. Dis..

[15] Boyett M.R., D’Souza A., Zhang H., Morris G.M., Dobrzynski H., Monfredi O. (2013). Viewpoint: Is the Resting Bradycardia in Athletes the Result of Remodeling of the Sinoatrial Node Rather than High Vagal Tone?. J. Appl. Physiol..

[16] Zdravkovic M., Perunicic J., Krotin M., Ristic M., Vukomanovic V., Soldatovic I., Zdravkovic D. (2010). Echocardiographic Study of Early Left Ventricular Remodeling in Highly Trained Preadolescent Footballers. J. Sci. Med. Sport.

[17] Luthi P., Zuber M., Ritter M., Oechslin E.N., Jenni R., Seifert B., Baldesberger S., Attenhofer Jost C.H. (2008). Echocardiographic Findings in Former Professional Cyclists after Long-Term Deconditioning of More than 30 Years. Eur. J. Echocardiogr..

[18] Coote J.H., White M.J. (2015). CrossTalk proposal: Bradycardia in the trained athlete is attributable to high vagal tone. J. Physiol..

[19] Katona P.G., Mclean M., Dighton DHGuz A. (1982). Sympathetic and parasympathetic cardiac control in athletes and nonathletes at rest. J. Appl. Physiol. Respir. Environ. Exerc. Physiol..

[20] Choudhury M., Boyett M.R., Morris G.M. (2015). Biology of the sinus node and its disease. Arrhythmia Electrophysiol. Rev..

[21] Taggart P., Critchley H., Lambiase P.D. (2011). Heart-brain interactions in cardiac arrhythmia. Heart.

[22] Heath B.M., Xia J., Dong E., An R.H., Brooks A., Liang C., Federoff H.J., Kass R.S. (1998). Overexpression of nerve growth factor in the heart alters ion channel activity and beta-adrenergic signalling in an adult transgenic mouse. J. Physiol..

[23] Du Y., Demillard L.J., Ren J. (2021). Catecholamine-induced cardiotoxicity: A critical element in the pathophysiology of stroke-induced heart injury. Life Sci..

[24] Zemtsovsky E.V.V., Bondarev S.A., Shulman V.A., Egorov D.F. (1995). Violation of the Function of the Sinus Node in Athletes. Sick Sinus Syndrome: Monograph.

[25] Mark RBoyett M.R., Yanni Y., Tellez J. (2021). Regulation of sinus node pacing and atrioventricular node conduction by HCN channels in health and disease. Prog. Biophys. Mol. Biol..

